# The Role of Probiotic Lactic Acid Bacteria and Bifidobacteria in the Prevention and Treatment of Inflammatory Bowel Disease and Other Related Diseases: A Systematic Review of Randomized Human Clinical Trials

**DOI:** 10.1155/2015/505878

**Published:** 2015-02-22

**Authors:** Maria Jose Saez-Lara, Carolina Gomez-Llorente, Julio Plaza-Diaz, Angel Gil

**Affiliations:** ^1^Department of Biochemistry & Molecular Biology I, School of Sciences, University of Granada, 18071 Granada, Spain; ^2^Institute of Nutrition & Food Technology “José Mataix”, Biomedical Research Center, University of Granada, 18100 Armilla, Spain; ^3^Department of Biochemistry & Molecular Biology II, School of Pharmacy, University of Granada, 18071 Granada, Spain

## Abstract

Inflammatory bowel disease (IBD), which includes Crohn's disease (CD) and ulcerative colitis (UC), is a chronic inflammation of the small intestine and colon caused by a dysregulated immune response to host intestinal microbiota in genetically susceptible subjects. A number of fermented dairy products contain lactic acid bacteria (LAB) and bifidobacteria, some of which have been characterized as probiotics that can modify the gut microbiota and may be beneficial for the treatment and the prevention of IBD. The objective of this review was to carry out a systematic search of LAB and bifidobacteria probiotics and IBD, using the PubMed and Scopus databases, defined by a specific equation using MeSH terms and limited to human clinical trials. The use of probiotics and/or synbiotics has positive effects in the treatment and maintenance of UC, whereas in CD clear effectiveness has only been shown for synbiotics. Furthermore, in other associated IBD pathologies, such as pouchitis and cholangitis, LAB and bifidobacteria probiotics can provide a benefit through the improvement of clinical symptoms. However, more studies are needed to understand their mechanisms of action and in this way to understand the effect of probiotics prior to their use as coadjuvants in the therapy and prevention of IBD conditions.

## 1. Introduction

Inflammatory bowel disease (IBD) can be defined as a disease of disrupted physiology, microbiology, immunology, and genetics [1]. IBD mainly includes Crohn's disease (CD) and ulcerative colitis (UC), which are characterized by chronic inflammation of the gastrointestinal tract. CD and UC differ by the intestinal localization and features of the inflammation. In this way, CD inflammation occurs anywhere in the gastrointestinal tract, whereas UC inflammation starts in the rectum and is restricted to the colon [1, 2].

Microorganisms in the human gut act in symbiosis to modulate different functions, such as the stimulation-regulation of epithelial innate immunity, the competitive exclusion of pathogens, and the production of important metabolites (i.e., carbohydrates, vitamins, and short chain fatty acids (SCFAs)) [3–5].

Traditional fermented products, breast milk, gastrointestinal tract content, and the feces of human subjects are the primary sources of LAB and bifidobacteria [6]. LAB and bifidobacteria produce lactic acid as a major metabolic end-product of carbohydrate fermentation and exhibit an increased tolerance to acidity. These bacteria contribute to the organoleptic and textural profile of many foods [7]. In addition to having important applications in the food industry, LAB and bifidobacteria can have beneficial health effects as an adjuvant to decrease the intestinal microbiota imbalance induced by the use of antibiotics or by pathological conditions, particularly IBD [5–11].

Probiotics are defined as “live microorganisms which, when administered in adequate amounts, confer a health benefit on the host” according to the consensus of a multinational expert group of scientists convened in 2001 by the Food and Agriculture Organization of the United Nations (FAO) [12]. The term synbiotic refers to a product that contains both probiotics and prebiotics. By understanding the mechanism of action of the bacterial strains that act as probiotics, it would be possible to define not only a specific and efficient therapy but rather an individual customized therapy to improve the specific disease symptoms and also restore the basic functioning of the gut. For this purpose, lactobacilli and bifidobacteria are the most widely used probiotics in humans.

The main aim of this work was to review the scientific evidence on the role of LAB and bifidobacteria, which are commonly used as probiotics, mainly in the prevention and treatment of IBD and other related IBD. In addition, we provide potential mechanisms of action of LAB and bifidobacteria in those conditions.

## 2. Methodology

**Figure 1:**
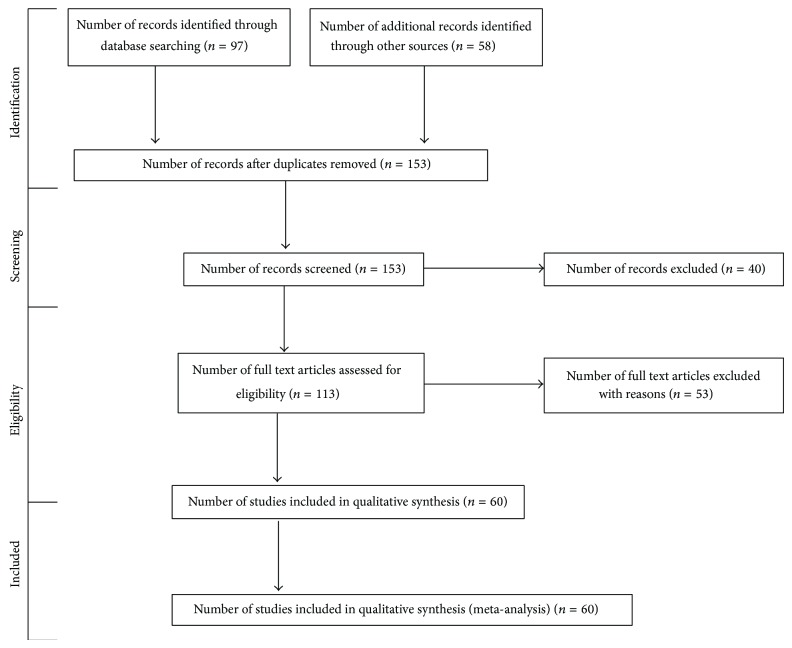


In this paper, we performed a systematic review of the role of fermented dairy products and LAB and bifidobacteria probiotics in the prevention and treatment of IBD. PubMed and Scopus were searched for human randomized clinical trials articles that were published between 1990 and June 2014 in English using the MeSH terms “dairy products” and “probiotics” combined with “inflammatory bowel disease,” “Crohn's disease,” and “ulcerative colitis.” Here, we evaluate results obtained using the following equation search (“dairy products” [MeSH Terms] OR (“dairy” [All Fields] AND “products” [All Fields]) OR “dairy products” [All Fields] OR (“dairy” [All Fields] AND “product” [All Fields]) OR “dairy product” [All Fields]) OR (“probiotics” [MeSH Terms] OR “probiotics” [All Fields]) OR (“microbiota” [MeSH Terms] OR “microbiota” [All Fields]) AND ((“inflammatory bowel diseases” [MeSH Terms] OR (“inflammatory” [All Fields] AND “bowel” [All Fields] AND “diseases” [All Fields]) OR “inflammatory bowel diseases” [All Fields] OR (“inflammatory” [All Fields] AND “bowel” [All Fields] AND “disease” [All Fields]) OR “inflammatory bowel disease” [All Fields]) OR (“colitis, ulcerative” [MeSH Terms] OR (“colitis” [All Fields] AND “ulcerative” [All Fields]) OR “ulcerative colitis” [All Fields] OR (“colitis” [All Fields] AND “ulcerative” [All Fields]) OR “colitis, ulcerative” [All Fields]) OR (“crohn disease” [MeSH Terms] OR (“crohn” [All Fields] AND “disease” [All Fields]) OR “crohn disease” [All Fields]) AND Clinical Trial [ptyp]). One hundred and thirteen original articles matching these criteria were initially selected, although only those articles that included specific LAB and bifidobacteria results (sixty) were later considered for the review and separated into four major topics: general aspects of probiotics in inflammatory bowel diseases, LAB, and bifidobacteria in Crohn's disease, in UC and on other inflammatory bowel diseases. In addition, we focused on the possible probiotic mechanism of action in IBD.  Figure 1 shows the flow diagram of searched articles [13] and Table 1 shows the summary of randomized clinical intervention trials of probiotics in IBD.

## 3. General Aspects of Probiotics in Inflammatory Bowel Disease

Nutrition seems to play a causal role in both UC and CD [14–17]. In this sense, in the past, IBD patients usually avoided dairy products to decrease disease symptoms [18]. However, currently, the recommendation is to have a complete and varied diet to prevent malnutrition, since a restrictive diet can lead to potential deficiencies in calcium, vitamin D, iron, vitamin B12, and *ω*-3 fatty acids, among other nutrients [19]. No specific diet has been shown to prevent or treat IBD. Only rather general statements have been done, and it seems that in genetically predisposed individuals, a high consumption of milk and other dairy products, as well as refined sugar and processed fat, may trigger the onset of IBD [16–21]. On the other hand, a diet rich in dietary fiber and fruits seems to be protective [20].

The efficacy of some probiotics to improve IBD patients' quality of life has been recently reported [22–28]. The human intestinal microbiota confers a multitude of important functions to the host, such as aiding in digestion or protecting from penetration by pathogenic microbes [29]. Moreover, microbial imbalance or dysbiosis, which is characterized by an increase in the harmful bacteria and a reduction in the levels of beneficial bacteria, is commonly associated with diseases such as IBD [30]. Both CD and UC are pathologies located in areas where there are high bacterial concentrations [10].

There is evidence that commensal enteric bacteria and their products create a local environment that affects the course of IBD [10]. These high bacterial concentrations in IBD patients are characterized by decreased numbers of LAB and bifidobacteria and increased numbers of* E. coli*, coliforms, and bacteroides in the colon [11]. In this sense, probiotics might increase intestinal biodiversity and improve the symptoms of IBD patients. Probiotics that may suppress inflammation and/or activate innate immunity could be used within therapeutic strategies to restore the host gut microbiota [31–33].

An individualized diet together with the use of a suitable probiotic may be the best strategy for improving IBD patients' quality of life. The specific knowledge of the mechanisms of action of probiotics would be a helpful tool to design an efficient and specific therapy to improve the specific disease symptoms in IBD.

Some of the proposed mechanisms by which probiotics may exert beneficial effects are (1) the production of SCFAs and lactate, which inhibit the growth of potentially pathogenic organisms and have an anti-inflammatory effect on the gut; (2) the increased transit time by the net flow of water from the blood to the intestinal lumen, which influences the adherence of bacteria to the intestinal wall; and (3) the reduced production of noxious substances that may contribute to the pathogenesis of IBD [34].

An altered epithelial barrier function contributes to intestinal inflammation. Moreover the gut microbiota plays a fundamental role in the maturation of the host's innate and adaptive immune responses [35]. The regulation of the host immune response by microbiota could involve toll-like receptors (TLR), since these receptors have also been shown to be an important link between innate and adaptive immunity through their presence in dendritic cells (DCs) and intestinal epithelial cells (IECs) [5, 36–38].

The induction of tolerance or intestinal inflammation depends on a host's ability to distinguish between pathogenic invaders and harmless resident organisms [36]. In IBD, patients seem to lose the normal human tolerance to commensal bacteria and their immune response is upregulated. Thus, TLRs recognize antigens from the microbiota as pathogens that are expressed by a variety of cells, including IEC and DCs [35]. TLR2 and TLR4 are involved in the maintenance of intestinal epithelial homeostasis [37]. In fact, a high expression of TLR2 and TLR4 is associated with IBD [5]. Pathogenic bacteria activate TLR4, enhancing barrier disruption, subsequently facilitating allergen translocation in the gut mucosa and the production of proinflammatory cytokines, such as tumor necrosis alpha (TNF-*α*), interleukin (IL)-1, and IL-6 [5, 35, 37, 38].

On the other hand, apical TLR9 activation in intestinal epithelial cells by* Lactobacillus rhamnosus GG* (LGG) prevents the degradation of I*κβ*-*α*, consequently suppressing nuclear factor kappa B (NF-*κ*B) activation and, in this way, preventing the production of proinflammatory cytokines [36, 38]. However, it is more complicated than that: genomic DNAs from* Bifidobacterium* and* Lactobacillus* strains interact with TLR2 and/or TLR9 to enhance the intestinal epithelial barrier function and to facilitate T_reg_ cell conversion via CD103+ DC [36, 37]. The interplay between microbiota and the gut immune system is complex.

Thus, Zeuthen et al. [37] reported that the combination of* L. acidophilus* X37,* L. paracasei* Z11,* L. casei* CRL431, LGG,* B. longum* Q46,* B. bifidum* Z9,* B. breve* 20091, and* B. bifidum* 20082a decreased IL-12 and TNF-*α* concentrations in culture supernatants and inhibited the Th1 skewing effect induced by strong stimulatory lactobacilli. This immunoinhibitory effect of bifidobacteria is TLR2-dependent and NOD2-independent [37]. Furthermore, a cell-free culture supernatant (CFS) from* Bifidobacterium breve* CNCM I-4035 also provides immunomodulatory effects on human intestinal DCs, mediated by cytokines [39, 40].

*Bacteroides* supports T helper (Th) and regulatory T (T_reg_) cell polarization in a TLR2-dependent manner through the recognition of polysaccharide A by DCs [36]. The short-term consumption of yogurt supplemented with* Lactobacillus strains* GR-1 and RC-14 promotes a desirable anti-inflammatory environment in patients that are consistent with the putative immunosuppressive role of the expanded CD4+CD25^high^  T cell population in humans [41]. Similarly, one study in mice described that probiotic bacteria (a mix of specific lactobacilli and bifidobacteria) may confer protection against chemically induced intestinal inflammation by T_reg_ cells through an immunoregulatory response involving IL-10 and transforming growth factor beta (TGF-*β*) [42]. Via both IL-10 production (which induces the differentiation of T_reg_) and direct interaction with IgA, probiotics attenuate the immune response against commensal bacteria [38]. More recently, Longhi et al. [43] described a human subpopulation of Th17 (supTh17) cells that are reduced in patients with IBD. This population of human supTh17 cells (in contrast to prototypic Th17) exhibits immune suppressive properties because it expresses high levels of both CD39 and FOXP3 and consequently produces extracellular adenosine. These differences suggest that supTh17 cells might be recruited as suppressor-type cells in the later steps on the immune response where these cells may help to resolve injury at specific sites [43].

In summary, a specific probiotic bacterial strain could improve the state of the intestine by facilitating epithelial barrier functions, inhibiting T_reg_ cell-mediated mucosal inflammation and increasing production of IL-10 and TGF-*β*. This inflammation reduction may prevent colitis. However, further research should be performed with new LAB strains in experimental models of IBD and humans with either CD or UC. Also, the use of combinations of different probiotics should be studied.

## 4. Role of Lactic Acid Bacteria and Bifidobacteria in Crohn's Disease

CD is a chronic inflammatory condition of the gastrointestinal tract driven by abnormal T cell responses to the intestinal microbiota [44]. Therapy often involves the induction of remission with corticosteroids and maintenance therapy with a combination of aminosalicylates and immunomodulators [45, 46]. Nevertheless, the importance of the intestinal microbiota in the etiology of mucosal inflammation provides a rationale for therapeutic strategies using probiotics and prebiotics in patients with CD [32].

Most of the published controlled trials showed that 5-aminosalicylic acid (5-ASA) is significantly more effective than placebo in preventing relapses of the disease. However, negative results have also been reported [47, 48]. Therefore, the prevention of relapses remains a major issue in the treatment of CD. The experimental and clinical data suggest that the intestinal bacteria may play a role in the postsurgical recurrence of CD. Consequently, the operated patient offers the best in vivo opportunity for assessing the influence of luminal microbiota on the occurrence of new lesions [49].

Prantera et al. [50] conducted a randomized, double-blind, controlled trial with LGG given immediately after all of the diseased gut was surgically removed. The basic idea of the study was that counterbalancing the harmful gut microbiota (a possible cause of recurrent lesions in CD) with a beneficial bacterium would prevent the appearance of lesions or reduce their severity. Forty-five patients were randomized to receive LGG or a placebo for 12 months. The results revealed no differences in endoscopic and clinical remission between the two groups [50]. In another similar study with fewer patients, Schultz et al. [51] also could not demonstrate a benefit of LGG in inducing or maintaining medically induced remission in CD [51].

The use of LGG is not restricted only to adult studies. Bousvaros et al. [52] conducted a randomized, double-blind, placebo-controlled trial to see if the addition of LGG to standard therapy prolonged remission in children with CD. Concomitant medications allowed in the study included aminosalicylates, 6-mercaptopurine, azathioprine, and low-dose alternate day corticosteroids. Seventy-five children with CD in remission were randomized to either LGG or placebo and followed for up to 2 years. The median time to relapse was 9.8 months in the LGG group and 11.0 months in the placebo group; 31% of the patients in the LGG group developed a relapse compared with 17% of the placebo group. However, these values were not significantly different [52]. The proposed explanation for these negative results was that patients with CD may be more resistant to colonization with this organism and thus might require a different dosage. Early endoscopic recurrence is frequent after intestinal resection for CD. Marteau et al. [53] tested* Lactobacillus johnsonii* LA1 in this setting with a randomized, double-blind, placebo-controlled study. Patients were randomized to receive two packets per day of lyophilized* L. johnsonii* LA1 or a placebo for 6 months, and no other treatment was allowed. The primary endpoint was endoscopic recurrence at six months, with a grade >1 in Rutgeerts' classification or an adapted classification for colonic lesions. Ninety-eight patients were enrolled (48 in the* L. johnsonii* LA1 group). At 6 months, endoscopic recurrence was observed in 64% of the placebo group and in 49% in the* L. johnsonii* LA1 group. The endoscopic score distribution did not differ significantly between the* L. johnsonii* LA1 and placebo groups. The* L. johnsonii* LA1 did not have a sufficient effect, if any, to prevent the endoscopic recurrence of CD [53].

Additionally, van Gossum et al. [54] evaluated the efficacy of oral administration of* L. johnsonii* LA1 on early postoperative endoscopic recurrence of CD. The oral administration of* L. johnsonii* LA1 in patients with CD failed to prevent early endoscopic recurrence at 12 weeks after ileocecal resection [54]. The use of individual LAB does not appear to produce clinical improvements in CD patients.

Probiotics differ strongly and it is not possible to extrapolate a positive or negative result from one strain to another strain. Therefore, the ineffectiveness of LGG in the study of Prantera et al. [50] cannot predict the inefficacy of* L. johnsonii* LA1 and cannot predict the inefficacy of other single strains in future trials [50]. Extrapolation of doses between various strains or products is also not possible. Mixtures of various strains could theoretically have additional or synergistic effects but they may also have antagonistic properties. Further studies of the microbiological, immunological, and clinical effects of lactic acid bacteria in maintaining disease remission are necessary.

Prebiotics have been associated with increased SCFA, mainly acetate, propionate, and butyrate [55]. Short-chain fatty acids, important nutrients for epithelial cells, are produced in the large bowel by the anaerobic bacterial fermentation of undigested dietary carbohydrates and fiber polysaccharides. Additionally, SCFA may actively contribute to the maintenance of colonic homeostasis [55].

A synbiotic is a regimen whereby probiotics are combined with prebiotics. Chermesh et al. [56] evaluated the use of Synbiotic 2000 in a clinical study to determine the efficacy in preventing the postsurgical recurrence of CD. Thirty patients were enrolled. No differences in either the endoscopic or the clinical relapse rate were found between patients treated with a once-daily dose of Synbiotic 2000 or a placebo. The Synbiotic 2000 had no effect on the postoperative recurrence of CD. The authors conclude that larger studies will be required because the number of patients may be too small to account for the individual differences in disease state, insufficient dosage, or negative interactions between specific probiotics and prebiotics. Additionally, using higher doses of a probiotic cocktail might prove effective [56].

Ten outpatients with active CD without a history of operation for CD were enrolled in a clinical study to evaluate the effects of synbiotics. Probiotics mainly comprised* Bifidobacterium* and* Lactobacillus*. Prebiotics, such as psyllium, are dietary substances that stimulate the growth and metabolism of protective commensal enteric bacteria. Patients were free to adjust their intake of probiotics or prebiotics throughout the trial. The Crohn's disease activity index (CDAI), International Organization for the Study of Inflammatory Bowel Disease (IOIBD) score, and blood sample variables were evaluated and compared before and after the trial. By the end of therapy, each patient had taken a 4.5 ± 2.4 × 10^10^ colonic forming-unit (CFU) daily probiotic dose, with six patients taking an additional 7.9 ± 3.6 g daily psyllium dose. Seven patients had improved clinical symptoms following combined probiotic and prebiotic therapy. Both CDAI and IOIBD scores were significantly reduced after therapy. There were no adverse events [55]. This study confirmed that high-dose probiotic and prebiotic cotherapy can be safely and effectively used for the treatment of active CD.

Finally, Steed et al. [57] evaluated synbiotic consumption in active CD. Thirty-five patients with active CD were enrolled in a randomized, double-blind, placebo-controlled trial, using a synbiotic comprising* Bifidobacterium longum* and Synergy 1. Their clinical status was scored and rectal biopsies were collected at the start, then again at 3- and 6-month intervals. The transcription levels of immune markers and mucosal bacterial 16S rRNA gene copy numbers were quantified using real-time PCR. Significant improvements in clinical outcomes occurred with synbiotic consumption, with reductions in both CDAI and histological scores. The synbiotic had little effect on mucosal IL-18, interferon *γ*, and IL-1*β*. However, significant reductions occurred in TNF-*α* expression in synbiotic patients at 3 months, but not at 6 months [57]. The synbiotic consumption was effective in introducing beneficial bacteria into the gastrointestinal tract in Crohn's patients, thereby modulating the species composition of the mucosal biofilm in the large bowel.

In conclusion, the investigation presented provides evidence that synbiotics (pre- and probiotics) have the potential to be developed into acceptable therapies for acute and active CD. More studies are needed to determine whether the synbiotic modulates other anti-inflammatory components of the mucosal microbiota [58, 59], or whether other synbiotic combinations can be as effective in CD [57].

## 5. Role of Lactic Acid Bacteria and Bifidobacteria in Ulcerative Colitis

UC is a nonspecific colorectal erosive inflammatory condition characterized by inflammation of the mucosa, erosion, and ulceration [60]. Patients with UC have periods of exacerbations and periods of remission. The treatment consists of inducing remission periods and maintaining those conditions using anti-inflammatory molecules (i.e., 5-ASA compounds); systemic and topic corticosteroids, immunosuppression drugs such as 6-mercaptopurine, and TNF-*α* antibodies have been used. However, these treatments present side effects that mean that a significant proportion of patients do not tolerate the existing treatments [23].

Numerous studies, in both IBD patients and gnotobiotic animals, have noted the influence of the intestinal bacteria on the development and/or exacerbation of UC [60]. Moreover, a lower number of bifidobacteria have been observed in the feces of UC patients than in healthy subjects [60]. Modulation of the intestinal microbiota can be performed either by antibiotics or by probiotics, but the former are not good candidates for chronic disease because of antibiotic resistance, potential side effects, and ecological concerns [61]. The modification of the intestinal microbiota through direct supplementation with protective bacteria could play a protective role in the inflammatory process [62].

Bifidobacteria-fermented milk (BFM) supplementation may reduce exacerbations of UC through the normalization of the intestinal microbiota [61]. Ishikawa et al. [60] reported that BFM supplementation reduced the luminal butyrate concentration, a key molecule in the remission of colitis. This reduction reflected the increased uptake or oxidation of SCFAs by the improved colorectal mucosa [60]. Similarly, Kato et al. [62] found increased levels of fecal butyrate, propionate, and SCFA acid concentrations in patients with active UC (mild to moderate), who received BFM together with conventional treatment [62]. In this pilot study, patients supplemented with BFM showed a significantly lower clinical activity index than the placebo group. Likewise, the posttreatment endoscopic index and histological score were reduced in the BFM group [62].

TNF-*α* exerts a pivotal role in the pathogenesis of active UC; therefore, inhibiting its secretion in inflamed UC mucosa is a major target for treating the disease and preventing relapse [63]. Coculturing colonic biopsies from active UC with* B. longum* reduced the release of TNF-*α* and IL-8 compared with the inflamed colonic tissue alone. It is well known that the activation of NF-*κ*B can regulate inflammatory cytokines such as TNF-*α*, IL-8, and IL-6. Immunohistochemical staining of NF-*κ*B p65 in colonic biopsies from active UC showed many cells with positive nuclear staining, whereas fewer NF-*κ*B-positive cells were found in the lamina propria after the tissues were cocultured with either* B. longum* or dexamethasone, which indicates that* B. longum* can inhibit NF-*κ*B activation in lamina propria cells [63].

Probio-Tec AB-25, a mixture of* L. acidophilus* strain La-5 and* B. animalis* subsp.* lactis* strain Bb-12, was tested for the maintenance of remission in patients with left-sided UC, in a 1-year, prospective, randomized, double-blind and placebo-controlled trial [64]. The safety and tolerance of Probio-Tec AB-25 and the placebo were good. Gastrointestinal symptoms were reported equally in both treatment groups and a relationship between Probio-Tec 25 and gastrointestinal side effects could not be established. At weeks 4 and 28, Bb-12 or La-5 were detected in 11 patients receiving probiotics. Five patients in the probiotic group (25%) and one patient in the placebo group (8%) maintained remission after 1 year of treatment. In the probiotic group, the median time to relapse was 125.5 days, versus 104 days in the placebo group. It is possible that in larger studies a significant difference could be achieved, but whether this would be of clinical significance is debatable [64].

The use of BIFICO (oral capsules of live enterococci, bifidobacteria, and lactobacilli) in combination with sulphasalazine (SASP) and glucocorticoid exerts some beneficial effects in preventing the relapse of UC [65]. The administration of BIFICO plus SASP and glucocorticoid to UC patients enlarged the number of bifidobacteria and lactobacilli and reduced the number of enterococci, bacteroides, and bifidobacteria present in the feces compared with the control group [65]. Moreover, Cui et al. [65] suggested that probiotics might block the activation of NF-*κ*B, decrease the expression of the proinflammatory cytokines TNF-*α* and IL-1*β*, and increase the expression of the anti-inflammatory cytokine IL-10 [64].

In the same way, the administration of* B. infantis* 35624 (1 × 10^10^ CFU) for six weeks to patients with mild- to moderate-active UC, during concurrent treatment with 5-ASA, significantly reduced plasma C-reactive protein (CRP) levels versus the placebo-treated controls [66]. However, when comparing pre- and posttreatment levels, there were no significant differences in the UC patients. Although CRP levels in the placebo group increased posttreatment, this result was likely because these patients did not receive steroid treatment during the trial period. In the case of plasma TNF-*α* levels, no significant differences were observed between the group that received the probiotic strain and the placebo group, or in the UC patients before treatment and after treatment. Regarding plasma IL-6, Groeger et al. [66] found a lower plasma level in UC patients compared with placebo controls; however, the authors did not find any change in the IL-6 levels in the UC patients between the pre- and posttreatment [66].

The most studied probiotic in clinical trials is* L. rhamnosus*, which is represented in the bowel of healthy individuals [67]. In agreement with this, Zocco et al. [67] studied the efficacy of LGG supplementation versus standard mesalazine for maintaining disease remission in UC patients. After 6 and 12 months of treatment the percentage of patients maintaining clinical remission was, respectively, 91% and 85% for the LGG group (1.8 × 10^10^ viable bacteria/day), 87% and 80% for the mesalazine group (2.400 mg/day), and 94% and 84% for the combined treatment (LGG plus mesalazine) [67].

The oral administration of Lacteol (Lacteol Fort, Rameda, Egypt), a probiotic preparation that contains 1 × 10^10^ CFU of* L. delbrueckii* and* L. fermentum*, together with 2,400 mg/day of sulfasalazine, during 8 weeks, to UC patients with chronic diarrhea, inhibited the extent of inflammation, prevented mucosal injury, and alleviated colitis [68]. One inflammatory cascade within the gut tissues during UC is characterized by the recruitment of circulating leukocytes and the release of proinflammatory mediators [68]. Lacteol administration not only reduced myeloperoxidase (MPO) activity, an index of leukocyte infiltration, but also reduced the colonic concentration of IL-6 and* TNF*-*α*. Regarding NF-*κ*B p65 levels, the UC patients showed the more activated NF-*κ*B p65 protein, whereas the lowest level was observed in the probiotic group [68].

In children with distal active UC, rectal administration of* L. reuteri* ATCC 55730 (as an enema solution containing 1 × 10^10^ CFU) for 8 weeks in addition to standard oral mesalazine resulted in a significant decrease in the Mayo DAI score (Mayo Disease Activity Index-DAI) compared with the children that received the corresponding placebo. In addition, all of the children on* L. reuteri* had a clinical response, whereas only 53% of the children on the placebo responded. Clinical remission was achieved in 31% of the* L. reuteri* group and in no children of the placebo group. At the posttrial the rectal mucosal expression levels (determined by RT-PCR in biopsy samples) of* IL*-*10* were significantly increased, whereas a significant decrease was found in the levels of* IL*-*1β*,* TNF*-*α*, and* IL*-*8*, only in the* L. reuteri* group [69].

Additionally, D'Incà et al. [70] evaluated the effect of an 8-week oral and/or rectal administration of* L. casei* DG on colonic-associated microbiota, mucosal cytokine balance, and TLR expression in patients with mild left-sided UC. The patients were divided into three groups: the first group received oral 5-ASA alone, the second group received oral 5-ASA plus oral* L. casei* DG (8 × 10^8^ CFU), and the third group received oral 5-ASA and rectal* L. casei* DG (8 × 10^8^ CFU). A significant improvement of the histological disease severity scores was found in patients receiving the probiotic strain by the oral or rectal route of administration. Nevertheless, oral supplementation with* L. casei* DG did not have a significant effect on the counts of* Enterobacteriaceae* or* Lactobacillus*. However, the occurrence of* Lactobacillus* and* Enterobacteriaceae* cultured from biopsy specimens was increased and decreased, respectively, in the group that took the probiotic rectally. Moreover, the rectal administration of* L. casei* DG significantly reduced* TLR*-*4* and* IL*-*1β* levels and significantly increased mucosal IL-10 [70].

Probiotic therapy can be improved through combination with a prebiotic (a nondigestible oligosaccharide that is absorbed in the upper gut). This combination is known as a synbiotic [71]. In a double-blinded randomized controlled trial, Furrie et al. [71] demonstrated that the administration of a synbiotic (*B. longum* plus Synergy 1), for a period of one month to patients with active UC, improved the full clinical appearance of chronic inflammation [71]. In this sense, the proinflammatory cytokines TNF-*α* and IL-1*α* were significantly reduced after treatment. In addition, the levels of bifidobacteria, determined by quantitative PCR, increased 42-fold in the synbiotic group but only 4.6-fold in the placebo group [71].

From this study, it is clear that synbiotic positively affects the chronic inflammation associated with UC. The comparison of the effectiveness of probiotics or prebiotics with that of synbiotic therapy was conducted by Fujimori et al. [72]. They designed a randomized trial to evaluate the effects of a 4-week treatment with probiotics, prebiotics, or synbiotics in patients with UC in remission. The probiotic group received 2 × 10^9^ CFU of* B. longum* (Bificolon, Nisshin Kyorin Pharmaceutical Co., Ltd., Tokyo) once daily; the prebiotic group was prescribed 4.0 g of psyllium to be taken twice daily. The synbiotic group simultaneously underwent probiotic and prebiotic therapies. The doses of aminosalicylates and prednisolone for UC treatment remained the same throughout the trial in all groups [72]. At the end of the trial, the authors found a statistically significant improvement of the Inflammatory Bowel Disease Questionnaire (IBDQ) scores in the synbiotic group. However, in this open-label trial, the authors did not perform a standard evaluation of the disease activity (endoscopic or histological evaluation) [72].

The beneficial effects of live* Bifidobacterium breve* strain Yakult (BbY) and galactooligosaccharide (GOS), as a synbiotic, were evaluated by Ishikawa et al. [73]. Patients diagnosed with UC received 1 g of the freeze-dried powder containing BbY (1 × 10^9^ CFU/g) and 5.5 g of GOS once/day. The control group comprised patients treated as usual (salazosulfapyridine, mesalazine, and steroids). After one year of intervention, the endoscopic scores of the synbiotic group were significantly lower than in the control group. In addition, the amounts of MPO in the lavage solution significantly decreased in patients with active UC after synbiotic treatment. Fecal bacteria analyses showed significant differences in the number of* Bacteroidaceae* before and after the synbiotic treatment in UC. Moreover, fecal pH was significantly lower after the synbiotic treatment [73].

The probiotic preparation VSL#3 has been extensively used. VSL#3 contains four strains of* Lactobacillus* (*L. casei*,* L. plantarum*,* L. acidophilus*, and* L. delbrueckii* subsp.* bulgaricus*), three strains of* Bifidobacterium* (*B. longum*,* B. breve*, and* B. infantis*), one strain of* Streptococcus salivarius* subsp.* thermophilus*, and cornstarch. VSL#3 is capable of colonizing the gut and significantly decreases fecal pH in UC patients that are intolerant or allergic to 5-ASA [74]. Furthermore, the intake of the probiotic mixture maintained remission in the great majority of UC patients that were intolerant or allergic to 5-ASA [74]. Additionally, it has been reported that balsalazide provides a more rapid relief of UC symptoms and induces complete remission in a greater percentage of patients than mesalamine, but these results were obtained using a high dose of balsalazide [75]. Balsalazide is converted into 5-ASA and 4-aminobenzoyl-*β*-alanine by the colonic bacteria. The use of 2.25 g of balsalazide (containing 750 mg of balsalazide disodium) plus 3 g of VSL#3 achieved remission faster than balsalazide or mesalazine. Moreover, balsalazide plus VSL#3 showed significant superiority in improving well-being and bowel frequency and endoscopic and histological scores were significantly better in the group of patients who received balsalazide/VSL#3 compared with the patients who received mesalazine at the end of the treatment [75]. Tursi et al. [75] showed that the combination of low-dose balsalazide plus VSL#3 resolved the problem of taking several capsules of balsalazide in comparison with mesalazine capsules to achieve remission in UC patients [75]. Therefore, the combination of low-dose balsalazide and VSL#3 may be a good choice in the treatment of active mild-to-moderate left-side- or distal-ulcerative colitis versus balsalazide or mesalazine alone [75]. This combination acts in two different ways to cease inflammation: 5-ASA inhibits some key enzymes of the inflammatory cascade, such as cyclooxygenase, thromboxane-synthetase, and platelet associated factor-synthetase and also inhibits the production of IL-1 and free radicals, whereas the action of probiotics includes the production of antimicrobials, competitive metabolic interactions with proinflammatory organisms and the inhibition of the adherence and translocation of pathogens [75].

In addition to this study, Tursi et al. [61] conducted a multicenter, double-blind, randomized, placebo-controlled, parallel study in patients affected by relapsing mild-to-moderate UC being treated with 5-ASA and/or immunosuppressants at stable doses to assess the effects of VSL#3 supplementation. They showed that VSL#3 supplementation (3.6 × 10^12^ bacteria per day) for 8 weeks was safe and able to reduce the UCDAI (Ulcerative Colitis Disease Activity Index) scores. Moreover, VSL#3 improved rectal bleeding and seemed to reinduce remission in relapsing UC patients, although these parameters did not reach statistical significance [61].

Bibiloni et al. [76] described that treatment of patients with active (mild to moderate) UC, not responding to conventional therapy, and receiving VSL#3 3.6 × 10^12^ bacteria daily in two divided doses for 6 weeks, resulted in a combined induction of remission/response rate of 94% in patients who completed the study. It is important to highlight that the authors reported no adverse events other than mild bloating [76]. In addition,* S. salivarius* subsp.* thermophilus* and* B. infantis* were detected by PCR/denaturing gradient gel electrophoresis, in association with biopsies collected after (but not before) treatment with VSL#3 in the case of 3 patients in remission [76].

In addition, the efficacy of VSL#3 in the induction and maintenance of remission and their safety and tolerability in children has been evaluated in a prospective, 1-year (or until relapse), placebo-controlled, double-blind study conducted by Miele et al. [23]. Patients (age range: 1.7–16.1 years) with newly diagnosed UC received either VSL#3 (weight-based dose, range: 0.45–1.8 × 10^12^ bacteria/day) or an identical placebo associated with concomitant steroid induction treatment. Remission was achieved in 92.8% of the children treated with VSL#3 and IBD conventional therapy and in 36.4% of the patients treated with placebo and IBD conventional therapy. Furthermore, 21.4% of the patients receiving VSL#3 treatment and 73.3% receiving the corresponding placebo (both groups also received IBD conventional therapy) relapsed within 1 year of follow-up. Regarding the endoscopic and histological scores, at 6 months and 12 months, they were significant lower in the VSL#3 group. It is important to emphasize that no side effects or significant changes from baseline values in any of the laboratory parameters examined were reported that could be attributed to treatment with either VSL#3 or placebo [23].

In conclusion, the use of probiotics and/or synbiotics has a positive effect in the treatment of UC and in the maintenance of remission periods. Probiotics and/or synbiotics reduced the expression of proinflammatory cytokines such us TNF-*α* and enhanced the expression of anti-inflammatory cytokine such us IL-10, likely through the inhibition of NF-*κ*B activation.

## 6. Role of Lactic Acid Bacteria and Bifidobacteria in Other Related Inflammatory Bowel Diseases

### 6.1. Pouchitis

Pouchitis is a common troublesome condition in surgical patients with ileal-pouch-anal-anastomosis (IPAA) [24] and is a nonspecific idiopathic inflammation of the ileal reservoir [77]. The daily administration of 500 mL of a fermented milk product (Cultura) containing live* L. acidophilus* (La-5) and* B. lactis* (Bb-12) for 4 weeks increased the number of lactobacilli and bifidobacteria in the UC/IPAA patients and remained significantly increased one week after the intervention. Moreover, involuntary defecation, leakage, abdominal cramps the need for napkins, fecal number, fecal consistency, fecal mucus, and urge to evacuate stools were significantly decreased/improved during the intervention period in the UC/IPAA patients [24].

The effects of the administration of VSL#3 (6 g/day) on patients with antibiotic therapy-induced pouchitis in remission have been studied by Kühbacher et al. [78]. The authors conducted a double-blind, randomized, placebo-controlled clinical trial. They took biopsies before and two months after the initiation of VSL#3 or placebo treatment. The patients who received the probiotic mixture were in remission at the time of the second biopsy, while the patients who received a placebo exhibited clinical and endoscopic signs of recurrent inflammation. Furthermore, there was an increase in the bacterial richness and diversity of the pouch mucosal microbiota in the VSL#3 patients compared with both patients in remission before therapy and patients developing pouchitis while receiving the placebo. The authors also described an increase in* Enterobacteriaceae* within the mucosa during the VSL#3 treatment. This fact indicates that remission maintenance during probiotic therapy is associated with the restoration of parts of the normal pouch biota [78].

Similarly, oral administration of high doses of VSL#3 was effective in the treatment of active mild pouchitis. The authors reported that treatment with VSL#3 significantly improved clinical, endoscopic, and histologic parameters on the PDAI (Pouchitis Disease Activity Index), with complete remission in almost 70% of the patients [77]. The microbiologic study showed a significant increase in the fecal concentration of bifidobacteria, lactobacilli, and* S. thermophilus*; however, no modification of* Bacteroides*, clostridia, coliforms, and enterococci was found, suggesting that the beneficial effect was not mediated by the suppression of the endogenous microbiota. These data indicate that the efficacy of VSL#3 may be related to increased concentrations of protective bacteria and further support the potential role for probiotics in IBD therapy [77].

In addition to these studies, Pronio et al. [79] carried out an open-label study with IPAA performed for UC; the patients received VSL#3 (0.45 × 10^12^ bacteria/day) or no treatment (control group) for 12 months. The patients treated with the probiotic showed a slight but significant reduction in PDAI scores after 3 months of treatment compared with baseline. This difference was maintained at 6 and 12 months of follow-up. Moreover, the data obtained by Pronio et al. [79] showed that probiotic administration in patients with IPAA expanded regulatory cells in the pouch mucosa. This finding was associated with an increased expression of* Foxp3* mRNA, a transcription factor needed for the generation and function of regulatory CD4+CD12+T cells and CD4+CD25+T cells that control the immune response to self and foreign antigens and are involved in oral tolerance. Furthermore, tissue samples showed a significant reduction in* IL*-*1β* mRNA expression. The authors concluded that the administration of probiotics after IPAA in patients without signs or symptoms of acute pouchitis induces an expansion of the associated regulatory cells [79].

### 6.2. Irritable Bowel Syndrome

IBD and irritable bowel syndrome (IBS) can be considered as different pathologies. IBD is recognized as an organic bowel disorder while IBS is a functional bowel disorder, although some particular cases in both disorders may display similar symptoms. Therefore, distinguishing clinical manifestations may be sometimes difficult [80, 81]. IBS, or spastic colon, is a symptom-based diagnosis characterized by chronic abdominal pain, discomfort, bloating, and altered bowel habits where the diarrhea or constipation may be predominate, or they may alternate. Indeed, the onset of IBS is more likely to occur after an infection [82, 83]. For that reason, favoring appropriate environmental intestinal conditions could delay or even avoid the onset of IBS. Thus, although considered as different pathologies, some authors recognized an association between IBD and IBS.

Hong et al. [84] evaluated the effects of probiotic LAB and bifidobacteria by-fermented milk (specifically* Lactobacillus sp*. HY7801,* Lactobacillus brevis* HY7401, and* Bifidobacterium longum* HY8004) on seventy-four IBS patients through clinical parameters and ^1^H nuclear magnetic resonance- (NMR-) based metabolomics from peripheral blood. This study reported decreased glucose and tyrosine levels and increased lactate in sera of patients but not in healthy volunteers. They argued that this increase in lactate in blood might be caused by intestinal microbiota that produce lactate through fermentation because of increased populations of intestinal LAB after probiotic administration. They further related the low serum glucose levels to elevated glycolysis in the body's attempt to accommodate the higher energy demand caused by small nutrient absorption [84]. They also suggested that decreased tyrosine is related to hepatobiliary disease, one of the most common extraintestinal manifestations of IBD, because tyrosine metabolism occurs mainly in the liver [84].

Furthermore, Dughera et al. [85] confirmed that the administration of a synbiotic agent in patients with constipation-variant IBS improved intestinal function and ameliorated the disease clinical manifestations. The synbiotic preparation included strains of* Bifidobacterium longum* W11, one of the most representative species of gut microbiota, and oligosaccharides, which exert a positive effect on intestinal motility and favor the development of bifidobacteria in the gut lumen [85]. Although these two works suggest that probiotics combined with prebiotics exert beneficial effects on IBS symptoms, more studies are needed to clearly demonstrate a positive effect [84, 85].

### 6.3. Cholangitis

Cholangitis is an infection of the common bile duct, the tube that carries bile from the liver to the gallbladder and intestines. It is usually caused by a bacterial infection, which can occur when the duct is blocked, such as a gallstone or tumor. The infection causing this condition may also spread to the liver [86].

The effects of a probiotic mixture (specifically* L. acidophilus*,* L. casei*,* L. salivarius*,* L. lactis*,* B. bifidum*, and* B. lactis*) have been evaluated on the liver biochemistry or function and symptoms in primary sclerosing cholangitis (PSC) patients with IBD that were receiving ursodeoxycholic acid (UDCA) maintenance therapy [87]. The absence of any significant positive effects was attributed to the concurrent use of UCDA, the relatively small number of patients studied, or the relatively short duration of treatment [87]. Nevertheless, Shimizu et al. [88] found that the combination of immunosuppressive therapy and a probiotic (*L. casei* Shirota, 3 g/day) provided benefits for both IBD and PSC. They suggested that bacterial microbiota and gut inflammation are closely associated with the pathogenesis of IBD-related PSC. This suppression of bowel inflammation and maintenance of bacterial homeostasis may be important for treating PSC [88] and other pathologies in which the host's relationship with the intestinal microbiota is relevant.

These contradictory effects described in the literature suggest that additional studies are needed to determine the effects of probiotics as adjunctive therapy for those inflammatory conditions of the gut.

## 7. Conclusions and Further Directions

This review focused on the clinical evidences that support the use of LAB and bifidobacteria probiotics as a valuable coadjuvant therapeutic strategy for the prevention and treatment of diseases such as IBD. The current scientific evidences are more significant in UC than in CD. However, more detailed mechanistic studies on the effectiveness of probiotics in IBD are necessary to determine their potential beneficial effects. Therefore, more clinical trials with the use of appropriate molecular tools are necessary to determine which main outcomes and additional immune- and inflammation-associated variables are clearly influenced, and particularly the cause of these changes in the development of IBD.

For this reason, more randomized double-blind placebo-controlled multicenter trials with appropriate doses and LAB are needed. However, well before this stage, preliminary studies confirming the potential probiotics' mechanisms of action need to be done in cell and animal models.

The investigation of the interactions between the environment, the diet, and the host constitutes one of the major issues in the development of IBD. The incidence of chronic disease in the adult state is related to epigenetic changes that happen earlier in life. Major clinical trials should also study the mechanisms of action of probiotics using new molecular tools such as the study of the microbiota changes using massive parallel sequencing (MPS), metabolomics, transcriptomics, and proteomics analyses of biopsies.

Beyond understanding the molecular mechanisms, further studies to evaluate the best dose-response-effect of probiotics are recommended, including following up with patients after the probiotic intervention to evaluate the persistence of beneficial effects.

Finally, determining the effect of fermented dairy products on the development and maintenance of the disease will also require specific clinical trials.

## Figures and Tables

**Table 1:** Summary of randomized clinical intervention trials of probiotics in IBD.

	Reference	Type of study	Number of patients	Age of patients (years)	Characteristics of patients	Probiotic strain	Medication	Intervention time/dose	Form of administration	Main outcome
Crohn's disease (CD)	Prantera et al., 2002 [50]	RDBPCT	45	22–71	Patients with a complete resection of all diseased intestine	*Lactobacillus rhamnosus *GG	No	365 d/6 × 10^10^ CFU twice daily	Oral	No effects compared with placebo group
	Schultz et al., 2004 [51]	RDBPCT	11	—	Patients with moderate to active CD	*Lactobacillus rhamnosus *GG	Yes	183 d/2 × 10^9^ CFU per day	Oral	No effects compared with placebo group
	Bousvaros et al., 2005 [52]	RDBPCT	75	5–21	Patients on CD remission	*Lactobacillus rhamnosus *GG	Yes	730 d/1 × 10^10^ CFU twice daily	Oral	No effects compared with placebo group
	Marteau et al., 2006 [53]	RDBPCT	98	27–42	Patients that had undergone surgical resection	*Lactobacillus. johnsonii* LA1	Yes	183 d/2 × 10^9^ CFU twice daily	Oral	No effects compared with placebo group
	van Gossum et al., 2007 [54]	RDBPCT	70	18–65	Patients with an elective ileocaecal resection	*Lactobacillus johnsonii* LA1	No	84 d/1 × 10^10^ CFU per day	Oral	No effects compared with placebo group
	Chermesh et al., 2007 [56]	RDBPCT	30	25 (mean age)	Patients that had undergone surgery treatment	^*^Synbiotic 2000	Yes	730 d/1 × 10^10^ CFU per day	Oral	No effects compared with placebo group
	Fujimori et al., 2007 [55]	CS	10	27 (mean age)	Patients with active CD	^*^Synbiotic therapy	Yes	395 d/7.5 × 10^10^ CFU per day and 3.3 g of psyllium thrice daily	Oral	Synbiotic therapy was safely and effectively used to treat active CD
	Steed et al., 2010 [57]	RDBPCT	35	18–79	Patients with active CD	*Bifidobacterium longum* plus ^*^Synergy 1	Yes	183 d/2 × 10^11^ viable CFU and 6 g Synergy I twice daily	Oral	Synbiotic improved clinical symptoms in patients with active CD

Ulcerative colitis (UC)	Ishikawa et al., 2003 [60]	RCT	21	39–60	Patients on UC remission	^*^BFM	Yes	365 d/1 × 10^10^ CFU per day	Oral	BFM supplementation successfully maintained remission
	Kato et al., 2004 [62]	RPCT	20	32 (mean age)	Patients with active UC	^*^BFM	Yes	84 d/1 × 10^10^ CFU per day	Oral	BFM supplementation was more effective than conventional treatment alone
	Tursi et al., 2004 [75]	RCT	90	19–69	Newly diagnosed or recently relapsed mild to moderate UC	^*^VSL#3	Yes	56 d/3 × 10^11^ CFU per day	Oral	Balsalazide/VSL#3 was significantly superior to balsalazide alone and to mesalazine in obtaining remission
	Cui et al., 2004 [65]	RCT	30	—	Patients with active UC	^*^BIFICO	Yes	56 d/1.26 g per day	Oral	BIFICO administration impeded the activation of NF-*κ*B and elevated the expression of *IL-10 *
	Furrie et al., 2005 [71]	RCT	18	24–67	Patients with active UC	*Bifidobacterium longum *plus ^*^Synergy 1	Yes	28 d/2 × 10^11^ CFU and 6 g of Synergy 1 twice daily	Oral	Short-term treatment improved the full clinical appearance of chronic inflammation in patients with active UC
	Zocco et al., 2006 [67]	ROLT	187	33 (mean age)	Patients on UC clinical remission	*Lactobacillus. *GG	Yes	365 d/6 × 10^9^ CFU twice daily	Oral	*Lactobacillus *GG was not inferior to mesalazine and was significantly better at delaying relapses
	Fujimori et al., 2009 [72]	RCT	120		Patients on remission or with mildly active UC without a history of operation for UC	*Bifidobacterium longum *plus psyllium	Yes	28 d/2 × 10^9^ CFU per day and 4 g of psyllium twice daily	Oral	Synbiotic treatment improved the quality of life better than probiotic or prebiotic treatment
	Miele et al., 2009 [23]	RDBPCT	29	1.7–16.1	Children newly diagnosed with UC	^*^VSL#3	Yes	365 d/4.5 × 10^11^–1.8 × 10^12^ CFU per day	Oral	VSL#3 was safe and effective in children treated for active UC^**^
	Hegazy and El-Bedewy, 2010 [68]	RCT	45	47 (mean age)	Mild to moderate UC patients with chronic diarrhea	^*^Lacteol	Yes	56 d/1 ×10^10^ CFU per day	Oral	Supplementation with probiotics could be advantageous in preventing relapse of UC and maintaining remission
	Tursi et al., 2010 [61]	RDBPCT	131	47 (mean age)	Patients with mild to moderate relapsing UC	^*^VSL#3	Yes	56 d/1.8 × 10^12^ CFU twice daily	Oral	VSL#3 administration reduced the UCDAI scores in patients affected by relapsing mild-to-moderate UC
	D'Incà et al., 2011 [70]	RCT	26	—	Patients with mild left-side UC	*Lactobacillus casei *DG	Yes	56 d/8 × 10^8^ CFU twice daily	Oral and rectal	5-ASA plus rectally administered probiotic modified the colonic microbiota, reduced the expression of *TLR-4, IL-1β*, and increased *IL-10* mRNA
	Wildt et al., 2011 [64]	RDBPCT	32	≥18	Patients with UC in remission	^*^Probio-Tec AB-25	No	364 d/2.5 × 10^10^ CFU per day	Oral	Probio-Tec AB-25 was well tolerated
	Ishikawa et al., 2011 [73]	RCT	41	45.5 (mean age)	Patients with mild to moderate UC	*Bifidobacterium breve *strain Yakult plus GOS	Yes	365 d/1 × 10^9^ CFU thrice a day and 5.5 g of GOS once a day	Oral	Synbiotic administration can improve the clinical condition
	Oliva et al., 2012 [69]	RCT	31	7–18	Patients with mild to moderate UC	*Lactobacillus reuteris *ATCC 55730	Yes	61 d/1 × 10^10^ CFU per day	Rectal enema	Rectal infusion decreased the expression of proinflammatory cytokines and increased the expression of IL-10 in children

5-ASA: 5-aminosalicylic acid; BFM: bifidobacteria-fermented milk; CFU: colonic forming unit; CS: clinical study; d: days; GOS: galactooligosaccharide; IBS: irritable bowel syndrome; IL: interleukin; NF-*κ*B: nuclear factor kappa B; OPUM: open-label prospective uncontrolled multicenter study; RCT: randomized clinical trial; RDBPCT: randomized double-blind placebo-controlled trial; ROLT: randomized open-label trial; RPCT: randomized placebo-controlled trial, TLR: toll-like receptor; UCDAI: ulcerative colitis disease activity index.

^*^Description of the bacterial and prebiotic contents of each product Synbiotic 2000: *Pediococcus pentosaceus*, *L. raffinolactis*, *L. paracasei* subsp. *paracasei* 19, and *L. plantarum* 2362 (10^10^ CFU of each bacteria) and *β*-glucans, inulin, pectin, and resistant starch (2.5 g of each fermentable fiber). Synbiotic therapy: *Bifidobacterium breve* and *Lactobacillus casei* (3 × 10^10^ CFU/daily of each bacteria) and 1.5 × 10^10^ CFU/daily of *Bifidobacterium longum* plus 3.3 g of psyllium twice daily. Synergy I: Orafti, Tienen, Belgium. BFM: live Yakult strains of *Bifidobacterium breve*, *Bifidobacterium bifidum,* and *Lactobacillus acidophilus* YIT 0168 in at least 10^9^ per 100 mL bottle. VSL#3: *Lactobacillus casei*, *Lactobacillus plantarum*, *Lactobacillus acidophilus* and *Lactobacillus delbrueckii* subsp. *bulgaricus*, *Bifidobacterium longum*, *Bifidobacterium breve*, *Bifidobacterium infantis*, *Streptococcus salivarius* subsp. *thermophilus,* and cornstarch. BIFICO: bifid triple viable capsule (oral capsules of live enterococci, bifidobacteria, and lactobacilli). Lacteol: 1 × 10^10^ CFU of *Lactobacillus delbrueckii* and *Lactobacillus fermentum. *Probio-Tec AB 25: *Lactobacillus acidophilus* strain LA-5 and *Bifidobacterium animalis* subsp. *lactis* strain BB-12 (1.25 × 10^10^ of each bacteria). Synbiotic zir fos: *Bifidobacterium longum* W11 (5 × 10^9^ CFU) and Fos-Actilight (2.5 g). ^**^Relative Risk of relapse within 1 year of follow-up (RR: 0.32 CI: 0.025–0.773).
